# Clinical Utility of Dual-Energy CT for Detection, Characterization, and Staging of Lung Tumors: A Rapid Review

**DOI:** 10.3390/diagnostics16162611

**Published:** 2026-08-18

**Authors:** Hassibullah Sidiqy, Khalida Sidiqy, Claudia Raluca Mariean, Marian Pop

**Affiliations:** 1 “George Emil Palade” University of Medicine, Pharmacy, Science and Technology of Târgu Mureș, 540142 Târgu Mureș, Romania; 2 Doctari GmbH, 10247 Berlin, Germany; 3 Pathophysiology Department, “George Emil Palade” University of Medicine, Pharmacy, Science and Technology of Târgu Mureș, 540142 Târgu Mureș, Romania; 4 Department ME2, “George Emil Palade” University of Medicine, Pharmacy, Science and Technology, 540142 Târgu Mureș, Romania; 5 Department of Radiology, Mureș County Clinical Emergency Hospital, 540142 Târgu Mureș, Romania

**Keywords:** dual-energy CT, lung cancer, pulmonary nodule, radiomics, iodine mapping, non-small cell lung cancer

## Abstract

**Background/Objectives**: Lung cancer remains one of the leading causes of cancer-related mortality worldwide. Conventional computed tomography (CT) is the preferred imaging modality for evaluating pulmonary nodules because of its high spatial resolution; however, it primarily provides morphological information, including lesion size, shape, and density. Dual-energy CT (DECT), a more recent imaging technique, uses two different energy levels to enable material decomposition and quantitative parameter assessment. These parameters may provide additional information regarding tumor perfusion, vascularization, and tissue composition. This rapid review aimed to evaluate the current evidence regarding the clinical utility of DECT in the detection, characterization, and staging of lung tumors. **Methods**: This rapid review was conducted according to the Preferred Reporting Items for Systematic Reviews and Meta-Analyses (PRISMA) guidelines. A literature search was performed in the PubMed and Cochrane Library databases for studies published between 2005 and 2026. Studies were included if they evaluated the detection, characterization, or staging of lung tumors using quantitative DECT parameters. Case reports, editorials, duplicate studies, and studies without quantitative DECT data were excluded. Descriptive data analysis was performed using Microsoft Excel. **Results**: A total of 24 studies were included, comprising 18 retrospective (75%) and 6 prospective studies (25%). Only one study evaluated the role of DECT in lung tumor detection, demonstrating improved detection of mixed ground-glass nodules and invasive adenocarcinoma. Significant correlations were found between iodine uptake and tumor perfusion, highlighting the potential of DECT to improve differentiation between benign and malignant lesions. Several studies also demonstrated associations between DECT parameters and tumor biomarkers, including Ki-67 Proliferation Index (Ki-67) expression, Epidermal Growth Factor Receptor (EGFR) mutation status, Programmed Death-Ligand 1 (PD-L1) expression, and treatment response in non-small cell lung cancer. In addition, DECT provided complementary metabolic information regarding tumor malignancy and showed correlations between iodine uptake and fluorodeoxyglucose (FDG) parameters. Associations between iodine volume and tumor differentiation grade were also reported. One study demonstrated the potential role of DECT in tumor staging by predicting mediastinal lymph node metastasis. Across all included studies, iodine-based parameters (50%), radiomics and material decomposition parameters (16.67% each), and spectral attenuation parameters (12.50%) were the most frequently investigated DECT metrics. **Conclusions**: DECT appears to be a promising complementary imaging technique that provides quantitative perfusion-related and compositional surrogate information beyond the morphological assessment offered by conventional CT. However, the current evidence remains heterogeneous and is largely based on retrospective studies with relatively small patient cohorts. Larger prospective studies with standardized imaging protocols are necessary to further establish the clinical utility of DECT in lung tumors.

## 1. Introduction

Lung cancer is one of the most common malignancies worldwide and remains a leading cause of cancer-related mortality [1]. Emerging imaging techniques like DECT are needed for accurate diagnosis, staging, and appropriate lesion characterization [2,3].

Medical imaging plays a central role in the evaluation and management of lung cancer. Computed tomography remains the standard imaging modality for evaluating pulmonary nodules and lung tumors. In contrast, positron emission tomography/computed tomography (PET/CT) improves staging accuracy by combining anatomical and metabolic information [4]. Despite its high spatial resolution and widespread clinical use, conventional CT has limitations in distinguishing tissues with similar densities and provides limited functional assessment of pulmonary lesions [5].

In light of these aspects, interest has increased in CT-based imaging techniques capable of providing additional functional and quantitative information beyond conventional morphological assessment [6]. In recent years, DECT has emerged as an advanced imaging technique for lung cancer diagnosis and staging. Spectral imaging techniques have gained increasing interest in thoracic oncology, with numerous studies investigating the role of DECT in lung cancer assessment. By acquiring images at two different energy spectra, DECT enables material decomposition and spectral analysis. This approach provides additional quantitative parameters beyond those available with conventional CT, including iodine concentration, normalized iodine concentration, extracellular volume fraction (ECV), spectral attenuation slope, and effective atomic number (Zeff). DECT-derived quantitative parameters may serve as potential imaging biomarkers for tumor characterization and therapeutic response evaluation. In addition, quantitative analyses derived from DECT may provide insight into tissue composition, vascularity, and perfusion characteristics of pulmonary lesions, potentially improving lesion characterization and differentiation between benign and malignant nodules [7].

Existing evidence remains heterogeneous regarding acquisition protocols, feature extraction methods, radiomics algorithms, and the imaging parameters evaluated, limiting direct comparisons between studies and broader clinical implementation [8]. Despite promising preliminary findings, the clinical applicability of DECT-derived quantitative biomarkers remains incompletely established, and further evaluation is needed to confirm current results [9]. Furthermore, although DECT is progressively entering routine radiological practice, its precise role in lung cancer diagnosis, staging, and treatment monitoring continues to evolve [7].

Therefore, this rapid review aimed to evaluate the current evidence regarding the clinical utility of DECT in the detection, characterization, and staging of lung tumors, with particular emphasis on emerging applications related to molecular profiling and treatment-response assessment as components of comprehensive tumor characterization and clinical management.

## 2. Materials and Methods

### 2.1. Study Design

This rapid review was reported in accordance with the Preferred Reporting Items for Systematic Reviews and Meta-Analyses (PRISMA) 2020 statement. The review was designed to provide a timely and structured synthesis of the current evidence regarding the clinical applications of DECT in lung cancer imaging. Given the rapidly expanding literature on DECT applications in thoracic oncology, a rapid review methodology was considered appropriate to provide a focused and up-to-date overview of the available evidence. Therefore, several methodological simplifications were adopted compared with a full systematic review. These included restricting the literature search to two electronic databases (PubMed and the Cochrane Library), including only English-language publications, not searching the grey literature, and not performing a meta-analysis because of the methodological heterogeneity of the included studies.

DECT is an advanced imaging technique that acquires CT data at two different X-ray energy levels, enabling material decomposition and the generation of quantitative imaging biomarkers, such as iodine concentration, Zeff, and virtual monoenergetic images (VMI). This rapid review aimed to summarize the current evidence regarding the clinical utility of DECT for the detection, characterization, molecular profiling, and staging of pulmonary nodules.

### 2.2. Search Strategy

**Figure 1:**
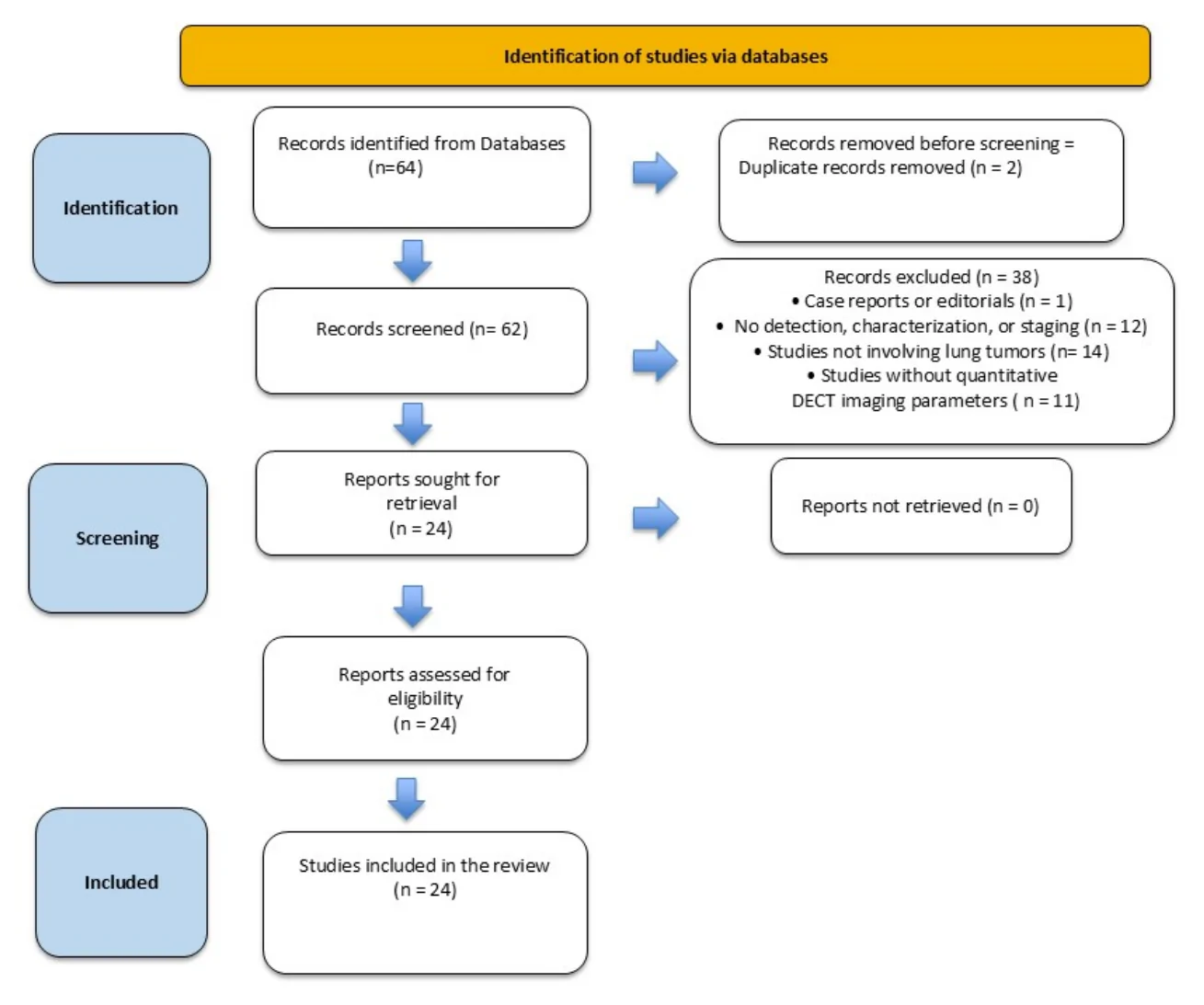
PRISMA flow diagram illustrating the study selection process for the rapid review of dual-energy CT in lung cancer imaging.

PubMed (National Library of Medicine) and the Cochrane Library (Wiley) were systematically searched in April 2026 to identify English-language studies published between January 2005 and March 2026. The search strategy combined free-text terms related to lung cancer and dual-energy CT. The complete search strategies, including Boolean operators, search fields, and database-specific syntax, are provided in Supplementary Table S1. Titles and abstracts were screened for relevance, followed by full-text assessment of potentially eligible articles. Reasons for exclusion at the title and abstract screening stage are presented in the PRISMA flow diagram (Figure 1); all reports sought for retrieval fulfilled the eligibility criteria at full-text assessment.

### 2.3. Inclusion Criteria

Studies were included in the current review if they met the following criteria:

• Prospective or retrospective studies investigating lung cancer using DECT or spectral CT;
• Studies evaluating the role of DECT in the detection, characterization (including molecular profiling and treatment-response assessment), or staging of lung tumors;
• Studies reporting quantitative DECT parameters, including iodine concentration, normalized iodine concentration, extracellular volume fraction, spectral attenuation slope, effective atomic number, virtual monoenergetic imaging, or radiomics features;
• Full-text articles available in English.

### 2.4. Exclusion Criteria

The following exclusion criteria were applied:

• Case reports, reviews, letters, editorials, conference abstracts, or non-original articles;
• Studies not involving lung tumors or pulmonary nodules;
• Studies lacking quantitative DECT imaging data;
• Duplicate publications.

### 2.5. Study Selection and Data Extraction

Study selection was performed according to predefined eligibility criteria.

Titles and abstracts were screened for relevance, followed by full-text review of potentially eligible studies. Study selection and data extraction were subsequently performed independently by two reviewers using a predefined data extraction form, with any disagreements resolved through discussion until consensus was reached.

The extracted information included study design, patient population, clinical objective, DECT parameters evaluated, and the main study findings. The included studies were also independently classified according to their primary objective and the quantitative DECT parameters investigated, as presented in Table 1 and Table 2. The study selection process is illustrated in the PRISMA flow diagram (Figure 1).

Extracted variables included study characteristics (author, year of publication, and study design), patient demographics, evaluated DECT parameters, and imaging findings related to lung cancer detection, characterization, staging, molecular profiling, and treatment response.

Extracted data were summarized in Table 1 to compare the diagnostic performance and potential clinical value of DECT across the included studies. Given the rapid review design, the broad scope of the review, and the methodological heterogeneity of the included studies, a formal risk-of-bias or certainty-of-evidence assessment was not performed. Therefore, the findings presented in this review should be interpreted as a descriptive synthesis of the currently available evidence rather than as a quantitative evaluation of study quality or certainty of evidence.

## 3. Results

### 3.1. Study Selection and Characteristics

This rapid review included 24 studies evaluating the use of DECT across various aspects of thoracic oncology, including the characterization, detection, and staging of lung tumors. The selected articles were categorized by study design into 18 retrospective studies (75%) and 6 prospective studies (25%).

#### 3.1.1. The Study Field of the Included Studies

The majority of studies (22/24, 91.7%) focused on broad tumor characterization, including conventional lesion characterization, molecular profiling, and treatment-response assessment. Only one study investigated the use of DECT for tumor detection, while another evaluated its role in staging by predicting lymph node metastasis.

#### 3.1.2. Analyzed Parameters

Regarding imaging biomarkers, iodine-based spectral parameters were the most frequently evaluated. Twelve studies (50%) investigated the potential association between iodine-based parameters and tumor vascularization and perfusion. Radiomics analysis was performed in 4 studies (16.67%), primarily to predict molecular characteristics, including EGFR mutation status and tumor invasiveness.

Additionally, material decomposition parameters were investigated in four studies (16.67%) and spectral attenuation parameters in three studies (12.50%), while extracellular volume (ECV) measurements were evaluated in one study (4.17%). Studies were assigned to a single category according to the predominant DECT parameter evaluated, as presented in Table 2.

### 3.2. Lesion Detection

A single study investigated the use of DECT for improving the detection of lung lesions. The study aimed to determine whether spectral imaging could improve lesion visibility and characterization compared with conventional CT. Zhang et al. showed that electron-density images improved visualization of small solid components within known ground-glass nodules, leading to the reclassification of some nodules from pure to mixed ground-glass [2].

### 3.3. Tumor Characterization Using Quantitative DECT Biomarkers

#### 3.3.1. Vascularization and Perfusion-Related Biomarkers

While only a limited number of studies investigated qualitative parameters derived from DECT images, most studies focused on quantitative biomarkers. Overall, 22 of the 24 included studies (91.67%) investigated the role of spectral imaging biomarkers in evaluating tumor vascularization, differentiation, proliferation, and molecular characteristics. These studies benefited from spectral CT’s unique capability to provide functional and compositional information in addition to the anatomical information obtained with conventional CT.

Among all investigated biomarkers, iodine concentration was the most frequently evaluated parameter (50% of cases), owing to its close relationship with tumor perfusion and vascularity. Chen et al. reported that iodine uptake measured with DECT was positively correlated with CT perfusion parameters in patients with lung cancer, suggesting that iodine-based spectral imaging may serve as an alternative imaging biomarker for assessing tumor blood flow and microvascular density [13]. In addition, Andersen et al. demonstrated a moderate correlation between iodine concentration measured with DECT and FDG uptake assessed by PET/CT in non-small cell lung cancer (NSCLC), suggesting that metabolic activity and tumor perfusion reflect different aspects of tumor biology [19]. In contrast, Kupik et al. reported no significant correlation between DECT and FDG PET/CT measurements in lung tumors and metastatic lymph nodes, suggesting that the two imaging modalities assess distinct biological characteristics of lung tumors [17].

#### 3.3.2. Differentiation of Benign and Malignant Pulmonary Lesions

He et al. investigated which quantitative DECT parameters could help differentiate malignant from benign solid pulmonary nodules. Venous-phase iodine concentration and normalized iodine concentration demonstrated area under the curve (AUC) values close to 0.89, suggesting that iodine-based spectral parameters may play an important role in differentiating malignant nodules [3].

Li et al. evaluated the ability of kilovolt peak (kVp)-switching dual-energy spectral CT to differentiate malignant from benign necrotic lung lesions. Spectral attenuation parameters and iodine-based measurements demonstrated higher diagnostic accuracy in distinguishing benign from malignant lesions, supporting the potential clinical value of DECT in diagnosing pulmonary lesions [18].

Several additional studies explored the value of spectral CT parameters in the differential diagnosis of malignant and benign pulmonary lesions. Jiang et al. found that ECV differed between lung cancers and benign lung lesions and showed moderate discriminatory performance. The highest diagnostic accuracy was achieved by combining conventional CT characteristics with spectral parameters [22]. Quantitative assessment of the extracellular space may provide additional information beyond the morphological features observed on conventional CT.

Zegadło et al. investigated the use of virtual monoenergetic imaging combined with iodine maps for evaluating solitary pulmonary nodules (SPNs). The authors concluded that VMI significantly increased SPN contrast while providing additional vascular detail, potentially improving lesion characterization and malignancy detection [15]. In addition, a study published by Ko et al. reported that spectral detector CT may improve the characterization of solitary pulmonary nodules by integrating iodine-related and spectral imaging characteristics, thereby enhancing the differentiation between benign and malignant lesions [24].

#### 3.3.3. Tumor Differentiation and Histological Grading

Several studies investigated the relationship between DECT parameters and tumor differentiation or histological grade. Iwano et al. assessed iodine distribution within lesions using three-dimensional iodine mapping on spectral CT and observed differences in iodine distribution among tumors with different histological grades. These findings suggested that spectral CT may reflect differences in tumor microvascular structure according to histological differentiation. In addition, lower iodine concentration was associated with poorer tumor differentiation [12].

More recently, Yu et al. found significant differences in virtual monoenergetic CT values and effective atomic number between low- and high-grade T1 lung adenocarcinomas. In contrast, iodine concentration, normalized iodine concentration, iodine concentration difference, and spectral attenuation slope did not differ significantly between pathological grades [31]. Furthermore, Deniffel et al. evaluated whether DECT-derived parameters, including IC and Zeff, could differentiate primary lung cancer from pulmonary metastases. The results demonstrated that combining DECT parameters with conventional CT attenuation values improved the diagnostic accuracy for distinguishing between these two types of pulmonary tumors [16].

#### 3.3.4. Tumor Proliferation and Growth Activity

The relationship between DECT parameters and tumor proliferation markers has also been investigated. Tian et al. reported that quantitative spectral CT parameters were significantly correlated with Ki-67 expression levels in patients with NSCLC [20]. Similarly, Su et al. demonstrated correlations between multiparametric DECT biomarkers and the Ki-67 proliferation index in lung adenocarcinoma [30]. These findings suggest that spectral CT biomarkers may provide indirect insight into tumor growth activity and proliferative potential.

#### 3.3.5. Radiomics and Prediction of Molecular Tumor Characteristics

In recent years, an increasing number of studies have explored the application of radiomics analysis using DECT data to predict molecular tumor characteristics. Jin et al. developed a DECT-based radiomics model to predict EGFR mutation status in NSCLC patients, demonstrating strong predictive performance [29]. Similarly, Ma et al. reported that spectral CT radiomics features could be used for predicting EGFR mutation status in lung adenocarcinoma (LUAD) [25].

More recently, Yamagata et al. investigated the association between DECT-derived iodine parameters and PD-L1 expression, concluding that spectral imaging may serve as a non-invasive imaging biomarker for predicting immunotherapy-related molecular characteristics [28]. Zheng et al. demonstrated that the invasiveness of lung adenocarcinoma presenting as ground-glass nodules (GGNs) could be predicted using a DECT radiomics model [23]. Likewise, Chang et al. showed that preoperative tumor invasiveness in lung adenocarcinoma could be predicted using multimodal spectral CT radiomics models [21].

#### 3.3.6. Differentiation of Specific Tumor Entities

In addition to molecular characterization, DECT has also been investigated for differentiating specific tumor entities. Winkelmann et al. evaluated the potential of single-phase DECT for differentiating pulmonary hamartomas from malignant lung tumors by analyzing spectral attenuation characteristics and iodine distribution within lesions [26].

#### 3.3.7. Therapy Response Assessment

This review also identified emerging applications of DECT-derived iodine quantification for assessing therapy response. Baxa et al. demonstrated that therapy-induced changes in iodine concentration correlated well with metabolic response, as assessed by FDG PET, in patients with NSCLC [14]. Similarly, Kim et al. demonstrated the potential role of DECT in evaluating treatment response following anti-angiogenic therapy in patients with NSCLC [11].

### 3.4. Staging

Only one study included in this rapid review evaluated the role of DECT in lung cancer staging. Accurate assessment of mediastinal lymph nodes is essential in lung cancer management, as treatment decisions and prognosis are strongly influenced by nodal metastatic involvement.

Xie et al. investigated whether quantitative parameters derived from spectral detector CT (SDCT), combined with clinical patient data, could predict lymph node metastasis in NSCLC. The authors reported that combining lymph node size, spectral attenuation slope (λ), and normalized iodine concentration improved the prediction of nodal metastasis, suggesting a potential role for DECT in increasing the diagnostic accuracy of lymph node evaluation in patients with lung cancer [27]. Although these findings are promising, evidence regarding the role of DECT in lymph node staging remains limited, as only a single study addressing this application was identified and discussed in this review.

## 4. Discussion

The current literature review suggests that DECT has emerging applications in the detection, characterization, and staging of lung tumors. By providing functional and compositional information alongside conventional anatomical assessment, DECT may improve lesion evaluation through spectral imaging techniques, including iodine quantification, virtual monoenergetic imaging, and material decomposition.

Several studies have demonstrated that DECT may improve lesion visualization, particularly of small pulmonary nodules and ground-glass opacities, which can be challenging to identify with conventional CT alone. Parameters such as IC, NIC, and spectral attenuation characteristics may enhance lesion conspicuity by increasing the contrast between lesions and the surrounding lung parenchyma. In particular, spectral CT has shown improved visualization of mixed ground-glass nodules [2].

However, these findings should be interpreted within the context of the currently available evidence. Most studies were conducted in preselected patient cohorts with already identified lesions rather than in true screening populations. Therefore, the present clinical utility of DECT appears to lie mainly in the further characterization of indeterminate findings detected on conventional CT rather than in primary lesion detection or lung cancer screening [2].

A substantial proportion of the available literature focuses on quantitative DECT biomarkers for tumor characterization. Iodine-related parameters are commonly used as surrogate markers of tumor perfusion and vascularity and may provide indirect information about tumor biology. A previous study demonstrated correlations between DECT-derived iodine uptake and CT perfusion parameters [13]. In addition, a moderate association between iodine concentration and metabolic activity, as assessed with FDG PET/CT, has been reported, suggesting that DECT and PET/CT may provide complementary information reflecting vascular and metabolic tumor characteristics, respectively [19]. Nevertheless, these associations have not been consistently observed across all studies. In particular, the absence of significant correlations between DECT and FDG PET/CT measurements in primary tumors and metastatic lymph nodes has also been reported, indicating that these imaging modalities should not be considered interchangeable [17]. From our perspective, the inconsistent correlations reported between DECT-derived iodine parameters and FDG PET/CT should not necessarily be interpreted as contradictory findings. Rather, they may reflect the fact that these imaging techniques interrogate different aspects of tumor biology, with FDG PET/CT primarily reflecting glucose metabolism and iodine-based DECT parameters providing information related to tumor vascularity and perfusion. Consequently, DECT may be better regarded as a complementary rather than competing imaging modality within a multiparametric assessment of lung tumors. The variability among published studies may also reflect important methodological differences, including heterogeneous patient populations, variations in tumor histology and disease stage and differences in contrast administration and image acquisition protocols, as well as the use of different DECT platforms and quantitative analysis methods. These factors may substantially influence iodine quantification and should be considered when comparing results across studies.

Beyond functional characterization, DECT has also demonstrated potential for differentiating benign from malignant pulmonary lesions. Iodine-based parameters showed promising diagnostic performance across several studies [3,10,15,18,24]. Additional biomarkers, such as ECV, may further improve diagnostic accuracy [22]. Considering the limitations of conventional CT in morphologically indeterminate pulmonary nodules, DECT may help reduce diagnostic uncertainty and potentially decrease the need for invasive diagnostic procedures. However, these findings still require confirmation and validation in larger prospective studies.

It should also be noted that benign inflammatory or vascular lesions may demonstrate increased enhancement, potentially overlapping with malignant lesions and thereby limiting specificity [18]. Furthermore, iodine quantification may be influenced by several technical factors, including contrast timing, acquisition protocols, and reconstruction algorithms. These limitations, together with intrinsic tumor heterogeneity, require caution in interpreting DECT-derived biomarkers.

In addition to lesion differentiation, several studies investigated associations between DECT-derived parameters and tumor biology. Differences in iodine distribution and spectral characteristics among tumors with different histological grades have been reported [12,31]. Furthermore, correlations between DECT biomarkers and the Ki-67 proliferation index have been described, suggesting a potential role for DECT as a non-invasive imaging biomarker of tumor aggressiveness [20,30]. Nevertheless, these findings remain influenced by methodological variability and require further validation before routine clinical implementation.

More recently, attention has increasingly focused on radiomics approaches based on DECT data, aiming to correlate imaging features with molecular tumor characteristics. These findings are consistent with the broader trend toward multiparametric and personalized approaches in thoracic oncology, where imaging-derived biomarkers are increasingly integrated with histological, inflammatory, and clinical data [32]. In addition, studies have also investigated the prediction of EGFR mutation status, PD-L1 expression, and tumor invasiveness in lung adenocarcinoma [21,23,25,28,29]. Although these approaches appear promising, most currently available studies are retrospective, involve relatively small patient cohorts, and frequently lack external validation. In addition, radiomic features are highly dependent on segmentation methods and imaging acquisition parameters, which may limit reproducibility. As quantitative imaging techniques continue to evolve, establishing reliable reference values represents an important step toward the clinical interpretation of CT-derived quantitative biomarkers [33]. In parallel, standardized acquisition and reconstruction protocols are essential for improving their reproducibility across institutions and imaging platforms. Consequently, DECT-based radiomics currently remains primarily a research application. In our opinion, the future clinical impact of DECT may increasingly depend on integrating multiple quantitative imaging biomarkers rather than isolated parameters alone. Combining spectral imaging biomarkers with radiomics, artificial intelligence, and clinical data may support more comprehensive predictive models and personalized decision-making in lung cancer. However, standardized acquisition and reconstruction protocols, reproducible feature extraction, external validation, and prospective multicenter studies remain essential before these approaches can be translated into routine clinical practice. Compared with lesion characterization, evidence regarding the role of DECT in staging, particularly mediastinal lymph node assessment, remains limited. Although preliminary findings suggest that DECT-derived parameters may help predict lymph node metastasis, the currently available evidence is insufficient, and additional studies are needed to clarify its clinical value in this setting [27]. Beyond diagnostic applications, DECT has also been explored for assessing therapy response. Iodine-based parameters may detect early vascular changes during treatment, potentially preceding size-based changes defined by Response Evaluation Criteria in Solid Tumors (RECIST) [11,14]. However, this application remains exploratory and extends beyond the currently established diagnostic role of DECT.

Overall, the available evidence indicates that DECT is progressively evolving beyond conventional lesion characterization toward a comprehensive imaging biomarker platform. Although current evidence remains heterogeneous and is predominantly derived from retrospective studies, the consistent evaluation of quantitative spectral biomarkers across multiple clinical applications highlights the growing role of DECT in precision thoracic oncology. In our view, future investigations should prioritize prospective multicenter validation, standardized quantitative imaging protocols, and clinically applicable predictive models rather than identifying additional isolated imaging biomarkers.

Despite these promising results, several challenges currently limit the widespread implementation of DECT in routine clinical practice. Quantitative DECT biomarkers remain influenced by differences in acquisition protocols, contrast administration, image reconstruction algorithms, and vendor-specific technologies, limiting direct comparison between institutions. Furthermore, standardized acquisition protocols, universally accepted quantitative thresholds, and robust evidence regarding biomarker reproducibility have not yet been fully established. Practical implementation also depends on the availability of dedicated DECT scanners and post-processing software, which may vary across healthcare settings. From an economic perspective, evidence regarding the cost-effectiveness of DECT remains limited, and further studies are required to determine whether its additional quantitative information translates into measurable clinical and economic benefits. Finally, successful integration of DECT into routine diagnostic workflows will require standardized reporting recommendations, prospective multicenter validation, and clear evidence demonstrating incremental clinical value beyond conventional CT.

Nevertheless, this rapid review has several limitations. First, only studies published in English were included, potentially introducing selection bias. Second, substantial heterogeneity existed among the included studies regarding imaging protocols, DECT parameters, patient populations, and study endpoints, limiting direct comparison between studies. Third, most available studies were retrospective and involved relatively small patient cohorts. Finally, due to the rapid review design, a formal risk-of-bias assessment was not performed. Despite the methodological heterogeneity of the available literature, the current study emphasizes the expanding role of DECT as a promising non-invasive imaging approach with potential applications across multiple stages of lung cancer evaluation. In addition to the limitations described above, the rapid review methodology itself has inherent constraints. Compared with a full systematic review, rapid reviews typically employ a streamlined methodology to facilitate timely evidence synthesis. Consequently, although a structured search strategy based on the PRISMA framework was applied, the search was limited to two databases, only English-language publications were included, and no formal risk-of-bias assessment was performed. Therefore, some relevant studies may have been missed, and the findings should be interpreted in light of these methodological limitations. Another limitation of this review is the absence of a formal methodological quality or risk-of-bias assessment of the included studies. Consequently, the findings should be interpreted with caution, as the overall strength of the evidence may have been influenced by differences in study design, patient selection, imaging protocols, and outcome reporting. Future systematic reviews incorporating formal quality assessment tools may provide a more robust evaluation of the available evidence.

## 5. Conclusions

This rapid review suggests that DECT may provide clinically relevant information beyond conventional CT, particularly in the characterization of lung tumors. By evaluating iodine-related parameters and spectral imaging features, DECT may provide additional insight into tumor vascularity and tissue composition, potentially supporting the assessment of indeterminate pulmonary lesions. The currently available evidence is promising but remains heterogeneous. Most studies are retrospective, involve relatively small patient cohorts, and differ substantially in imaging protocols, acquisition techniques, and post-processing methods, thereby limiting the reported findings. Emerging applications, including radiomics analysis and therapy response assessment, further suggest that DECT may have broader future applications in thoracic oncology. However, these approaches remain largely investigational and are not yet ready for routine clinical implementation.

Overall, DECT is a promising complementary imaging technique for evaluating lung tumors. Nevertheless, its role in routine clinical practice has not yet been fully established and requires further validation through larger prospective multicenter studies with standardized imaging protocols and external validation.

## Figures and Tables

**Table 1:** Summary of Included Studies.

No.	Author (Year)	Study Design	Sample Size	DECT Parameters	Key Findings	Clinical Field
1	Chae (2008) [10]	Prospective	49 patients	Iodine-enhanced and Virtual Non-Contrast Imaging (VNC)	Improved assessment of lesion enhancement and intranodular calcifications	Characterization
2	Kim (2012) [11]	Prospective	10 patients	Iodine-enhanced imaging	Improved assessment of response to anti-angiogenic therapy	Therapy Response
3	Iwano (2015) [12]	Retrospective	60 patients	Three-dimensional iodine volume	Lower iodine concentration was associated with poor tumor differentiation	Characterization
4	Chen (2017) [13]	Prospective	28 patients	Iodine uptake, perfusion parameters	Demonstrated a significant correlation with perfusion CT parameters	Characterization
5	Baxa (2020) [14]	Prospective	45 patients	DECT iodine quantification	Demonstrated a correlation between iodine uptake and FDG uptake during staging and follow-up	Therapy Response
6	Zegadło (2020) [15]	Prospective	65 patients	Virtual Monoenergetic Imaging (VMI), iodine maps	Improved differentiation between benign and malignant solitary pulmonary nodules	Characterization
7	Deniffel (2021) [16]	Retrospective	168 patients	Iodine Concentration (IC), Zeff	Improved differentiation between primary lung tumors and pulmonary metastases	Characterization
8	Kupik (2021) [17]	Retrospective	68 patients	IC, normalized iodine concentration	No significant correlation between DECT iodine parameters and PET/CT findings	Characterization
9	Li (2021) [18]	Prospective	71 patients	Spectral slope, iodine concentration	Improved differentiation of necrotic pulmonary lesions	Characterization
10	Andersen (2022) [19]	Retrospective	22 patients	Iodine concentration	Provided complementary metabolic information compared with FDG uptake	Characterization
11	He (2022) [3]	Retrospective	147 patients (151 nodules)	IC, NIC, Zeff	Improved differentiation between malignant and benign pulmonary nodules	Characterization
12	Tian (2022) [20]	Retrospective	215 patients	IC, NIC, 40-keV VMI	Demonstrated a significant correlation with Ki-67 expression in non-small cell lung carcinoma (NSCLC)	Molecular Profiling
13	Chang (2023) [21]	Retrospective	176 patients	Multimodal spectral radiomics	Improved prediction of invasive lung adenocarcinoma	Characterization
14	Jiang (2023) [22]	Retrospective	128 patients	ECV, IC, NIC, Zeff	ECV improved differentiation between benign and malignant pulmonary lesions	Characterization
15	Zhang (2023) [2]	Retrospective	150 patients	Electron density imaging	Improved detection of mixed ground-glass nodules and invasive adenocarcinoma	Detection
16	Zheng (2023) [23]	Retrospective	92 patients	Radiomics, VMI	Improved prediction of ground-glass nodule invasiveness	Characterization
17	Ko (2024) [24]	Retrospective	71 patients	IC, NIC, spectral slope	Improved differentiation between benign and malignant pulmonary nodules	Characterization
18	Ma (2024) [25]	Retrospective	175 patients	DECT-derived radiomics and spectral parameters	Improved prediction of EGFR mutation status in lung adenocarcinoma	Molecular Profiling
19	Winkelmann (2024) [26]	Retrospective	46 patients (47 lesions)	Fat fraction, iodine density, VNC	Fat fraction showed the highest diagnostic accuracy for differentiating hamartomas from malignant lesions.	Characterization
20	Xie (2024) [27]	Retrospective	91 patients	NIC, spectral slope	Improved prediction of lymph node metastasis in NSCLC	Staging
21	Yamagata (2024) [28]	Retrospective	37 patients	Iodine density histogram	Improved prediction of PD-L1 expression status	Molecular Profiling
22	Jin (2025) [29]	Retrospective	115 patients	DECT-based radiomics model	Accurate prediction of EGFR mutation status	Molecular Profiling
23	Su (2025) [30]	Retrospective	79 patients	VNC, iodine parameters	Quantitative iodine parameters correlated with Ki-67 expression	Molecular Profiling
24	Yu (2025) [31]	Retrospective	161 lesions	Spectral CT parameters	Improved differentiation of pathological tumor grades	Characterization

Abbreviations: CT, computed tomography; DECT, dual-energy computed tomography; ECV, extracellular volume fraction; EGFR, epidermal growth factor receptor; FDG, fluorodeoxyglucose; IC, iodine concentration; Ki-67, Ki-67 proliferation index; NIC, normalized iodine concentration; NSCLC, non-small cell lung cancer; PD-L1, programmed death-ligand 1; PET/CT, positron emission tomography/computed tomography; VMI, virtual monoenergetic imaging; VNC, virtual non-contrast imaging; Zeff, effective atomic number.

**Table 2:** Distribution of DECT parameter categories across included studies.

Parameter Category	n	n (%)	Examples	Studies (Table 1)
Iodine-based	12	50.00%	IC, NIC, iodine uptake, iodine maps, iodine density	[1,2,3,4,5,6,8,10,11,12,21,23]
Radiomics	4	16.67%	Spectral/multimodal radiomics	[13,16,18,22]
Spectral attenuation	3	12.50%	Spectral slope, monoenergetic CT values	[9,17,20]
ECV	1	4.17%	ECV measurements	[14]
Material decomposition	4	16.67%	Zeff, electron density, fat fraction	[7,15,19,24]

Abbreviations: CT, computed tomography; DECT, dual-energy computed tomography; ECV, extracellular volume fraction; IC, iodine concentration; NIC, normalized iodine concentration; Zeff, effective atomic number.

## Data Availability

All data analyzed in this review are available in the included publications and are summarized in the article.

## Supplementary Materials

The following supporting information can be downloaded at https://www.mdpi.com/article/10.3390/diagnostics16162611/s1: Table S1. Complete search strategies used for the PubMed and Cochrane Library databases.

## Abbreviations

The following abbreviations are used in this manuscript:

AUC	Area under the curve
CT	Computed Tomography
DECT	Dual-Energy Computed Tomography
ECV	Extracellular Volume Fraction
EGFR	Epidermal Growth Factor Receptor
FDG	Fluorodeoxyglucose
GGN	Ground-Glass Nodule
IC	Iodine Concentration
LUAD	Lung Adenocarcinoma
LN	Lymph Node
kVp	Kilovolt peak
NIC	Normalized Iodine Concentration
NSCLC	Non-Small Cell Lung Cancer
PET	Positron Emission Tomography
PET/CT	Positron Emission Tomography/Computed Tomography
PD-L1	Programmed Death-Ligand 1
PRISMA	Preferred Reporting Items for Systematic Reviews and Meta-Analyses
RECIST	Response Evaluation Criteria in Solid Tumors
SDCT	Spectral Detector Computed Tomography
SCT	Spectral Computed Tomography
SPN	Solitary Pulmonary Nodule
VMI	Virtual Monoenergetic Imaging
VNC	Virtual Non-Contrast Imaging
Ki-67	Ki-67 Proliferation Index
Zeff	Effective Atomic Number
MeSH	Medical Subject Headings
MRI	Magnetic Resonance Imaging
