# Supplementary material for: Clinical Utility of Dual-Energy CT for Detection, Characterization, and Staging of Lung Tumors: A Rapid Review
Source: Diagnostics (Basel). 2026 Aug 18;16(16):2611. doi: 10.3390/diagnostics16162611 (PMC13511644; doi:10.3390/diagnostics16162611)
Supplement: Supplementary file 1 [file diagnostics-16-02611-s001.zip › diagnostics-4375427-supplementary.pdf]

Table S1. Complete search strategies used for the PubMed and Cochrane Library databases.

Database	Platform	Search date	Search strategy	Filters (publication language/publication period)
PubMed	National Library of Medicine	April 2026	("lung cancer"[Title/Abstract] OR "lung neoplasm*"[Title/Abstract] OR "pulmonary nodule*"[Title/Abstract]) AND ("dual-energy CT"[Title/Abstract] OR "dual-energy computed tomography"[Title/Abstract] OR "spectral CT"[Title/Abstract]) AND (detection OR characterization OR staging OR radiomics)	English; 1 January 2005–31 March 2026
Cochrane Library	Wiley	April 2026	("lung cancer" OR "lung neoplasm*" OR "pulmonary nodule*") AND ("dual-energy CT" OR "dual-energy computed tomography" OR "spectral CT") AND (detection OR characterization OR staging OR radiomics)	English; 1 January 2005–31 March 2026
